# Work life, relationship, and policy determinants of health and well-being among Filipino domestic Workers in China: a qualitative study

**DOI:** 10.1186/s12889-019-6552-4

**Published:** 2019-02-23

**Authors:** Brian J. Hall, Melissa R. Garabiles, Carl A. Latkin

**Affiliations:** 1 Global and Community Mental Health Research Group, Faculty of Social Sciences (E21), Psychology Department, University of Macau, Avenida da Universidade, Taipa, Macau, SAR People’s Republic of China; 2 Department of Psychology, School of Social Sciences, Ateneo de Manila University, Quezon City, Philippines; 3 Department of Health, Behavior and Society, Johns Hopkins Bloomberg School of Public Health, Baltimore, MD USA

**Keywords:** Migrant workers, Needs assessment, Mental health, Physical health, Domestic worker, Decent work

## Abstract

**Background:**

Overseas Filipino workers (OFWs) comprise one of the largest populations of migrant workers globally. Within China, they represent the largest group of imported domestic workers. Little is known about their working conditions or how this might affect their health and wellbeing.

**Methods:**

This qualitative study explored the working conditions and risk factors for poor health in a sample of temporary female Filipino domestic workers in Macao, China. Focus group discussions with female domestic workers (*n* = 22) and in-depth interviews with key informants (*n* = 7) were conducted.

**Results:**

Domestic workers reported physical (e.g., hypertension, chronic pain, diabetes, poor sleep), and mental health problems (depression, anxiety), and addictive behaviors (gambling, alcohol misuse), along with significant structural, linguistic, financial, and cultural barriers to healthcare access to address these concerns. Adverse working conditions including poor treatment and abuse by employers, lack of privacy and inadequate sleeping areas in employers’ homes or in crowded boarding houses, language barriers, inadequate and poor enforcement of labor protections, and discrimination. Domestic workers also cited exorbitant agency fees and remittances causing significant financial stress. Kinship network ties with family members back home were fraught with infidelity, difficulty parenting, misuse of remittances, and family misconceptions of domestic workers’ situation abroad. Lack of quality social support and peer social networks exacerbated these conditions.

**Conclusions:**

In this sample of Filipino migrant domestic workers, stressors experienced within the host country were commonly reported. Indebtedness and low salaries limits social mobility. Psychosocial and policy-level interventions are needed to improve the health and wellbeing of this population of migrant women.

## Background

Migration is increasing globally. In the past 10 years, the number of labor migrants has grown from 191,269,100 to 243,700,236 [36]. Unskilled and low wage workers are socially vulnerable and frequently report poor health, and mental health, and labor exploitation [7]. Female migrant domestic workers are particularly vulnerable since many work within private homes, isolated from community supports. It is difficult to monitor their health and well-being and to intervene on potentially abusive labor practices. The International Labor Organization [23] reported that domestic workers typically receive low salaries, work long hours, and have limited rest periods. Domestic workers are at risk for physical, mental, and sexual abuse. Their marginalized status presents numerous and compounding challenges to their well-being.

Labor migrants are exposed to various threats to health that can accrue during their pre-migration period, during migration, and after arrival in their destination country [25, 45]. Overseas Filipino workers (OFW) comprise a large population primarily motivated to leave home to seek employment abroad due to poverty and lack of job opportunities [13, 38]. According to the Survey on Overseas Filipinos, as of 2016, there were over 2.2 million OFWs globally [32]. Economic disadvantage in home countries is often accompanied by poor medical care, exposure to violence, and other traumas, which can decrease population health before leaving the country [19, 45].

Within their receiving country context, female migrant domestic workers may experience loneliness, lack of social networks or poor network support [12, 29], and stress related to living away from their families [17], which may exacerbate previously accumulated vulnerabilities. Within their work setting, they may be at risk for adverse occupational threats, including long working hours, abusive or exploitative relationships with their employers, and work related injuries [1]. These working conditions are known to exert significant influence on population health. The social determinants of health – broadly, working and living conditions, and social, economic, and cultural environments, can shape the distribution of migrant health [24, 28].

Poor mental health is a common complaint among labor migrants. A systematic review of 35 studies indicated that roughly 20% of labor migrants met the criteria for a common mental disorder such as anxiety and depression [27]. This is especially true among those whose jobs in receiving countries involved a decline in their socioeconomic position resulting from underemployment relative to their skills, expertise, or training [14]. In addition to poor mental health, stressors associated with migration and migrant work is also linked to poor health [8, 9, 18, 27, 33, 42–45]. For example, stressors from work, relationships, and daily living may lead to exhaustion, burnout, headache, and sleep difficulties [40].

Migrants experience key barriers to receiving mental and physical health care [2, 21, 26, 39, 41]. Examples include lack of finances, language differences, lack of insurance, lack of knowledge regarding health insurance coverage and healthcare systems, racism, and discrimination [2, 39]. Poor access to healthcare is itself a key determinant of worsening health conditions [41].

In the Macao Special Administrative Region (SAR), China, the context of the present study, Filipinos are the second largest group of non-resident workers and the largest population of non-Chinese migrant labor. As of September 2017, 13,535 Filipinos were reported to be employed as domestic workers (Macau Labour Affairs [11]). Domestic workers in Macao either live with their employers or live outside of their employer’s home, mostly in boarding houses. They migrate from the Philippines primarily to seek economic opportunities in order to support their families through remittances. They may live abroad for many years, without returning home or seeing their children and family more than once per year.

Through their labor, OFWs greatly contribute to the economic prosperity of many countries, and their financial remittances account for over 10% of The Philippines gross domestic product. Despite their importance and the size of this group, no previous study was conducted to understand their health needs. The current study aimed to identify key health issues migrant Filipino domestic workers were facing in their post-migration context, and the social determinants of these issues, in order to develop plans and policies to improve their health and well-being.

## Methods

### Data collection

Two types of interviews were conducted: Focus Group Discussions (FGDs) with female Filipino Domestic Workers (*n* = 5 FGDs, *n* = 22) and key informant interviews with local church leaders (*n* = 3), consulate office members (*n* = 2), and NGO field staff (*n* = 2). Given the gendered nature of the work involving cleaning, cooking, child and elder care, there are no known male domestic workers in Macao. Domestic worker participants were identified through social networks and existing relationships between the researchers and the community, and people supporting the community (Church leaders and local NGOs). Key informants were identified through existing relationships with the researchers. Key informants were trusted members of the local Filipino community (e.g., Priests and consulate staff) or local Chinese social workers who work in NGOs.

All participants were purposively sampled and contacted via face-to-face, telephone, and social media. Data collection took place from May to August 2015, in Macao (SAR), People’s Republic of China. Participants were told that the FGDs were about problems experienced by the community of Filipino domestic workers in Macao. They were assured of confidentiality and that their names would not be included in reports. Three domestic workers refused to participate because they were busy with work or leisure activities.

For the domestic worker FGDs, an interview guide was developed to assess community needs, following standard practices for field-based qualitative/ethnographic fieldwork [15]. A total of 7 open-ended questions were asked in reference to members of the domestic worker community in Macao (Table 1), which was pilot tested before the study began. The domestic workers were asked to report about their community rather than their own personal experiences, to increase the potential for the findings to generalize, and reduce concealment of sensitive issues owing to personal embarrassment or stigma.

**Table 1:** Interview questions

Interview Questions	
What are all the challenges that domestic workers in this community experience?	
How would you know if a domestic worker in the community is doing well? Describe this person.	
How would you know if a domestic worker in the community is not doing well? Describe this person.	
What are all the ways domestic workers in your community rely on one another?	
Name all of the people that members of the domestic worker community rely on for assistance.	
What are all the challenges that domestic workers in this community experience regarding health?	
What are all the challenges that domestic workers in this community experience in their relationships?	
*Additional Questions for Key informants:*	
Where do domestic workers go to receive healthcare and mental healthcare?	
What challenges do domestic workers face in accessing healthcare?	
What kinds of health-related programs might benefit this community?	

Probing follow-up questions were used following standard qualitative interviewing procedures [15]. The present study focused on responses characterizing the key health issues that Filipino domestic workers experience and the modifiable social determinants of health that drive these concerns.

The average length of a domestic worker FGD was 1.27 h (*SD* = 0.27), with key informant interviews lasting, on average 1.80 h (*SD* = .92). All FGDs and key informant interviews were audio recorded, and field notes were taken during interviews. All FGDs with female domestic workers were carried out by one doctoral-level female Filipino researcher (MRG), a university lecturer in psychology at the time of the study, with significant training and experience in qualitative interviewing. All interviews were conducted in Tagalog. The key informant interview with a Cantonese speaking key informant was conducted by a Cantonese native speaker with significant training in qualitative interviews. FGDs with Filipino domestic workers took place in community settings (e.g., NGO community center *n* = 2) or in participants’ homes (i.e., boarding houses *n* = 3). Key informant interviews took place within participants’ homes and NGO community centers, according to their preferences. All interviewees participated once. Research ethics approval was obtained by the Research Ethics Panel, University of Macau. All participants received informed consent procedures, and written consent was obtained for study participation. No compensation was offered for participation. Participants were informed that the project was being conducted with the support of a local NGO to identify key needs of the community and that the results would be disseminated to this NGO, and in academic venues.

### Analysis

Interviews were transcribed verbatim and checked against audio recordings for accuracy. All interviews were translated into English for analysis by MRG, who is fully bilingual in Tagalog and English, and who carried out the interviews. All names and identifying information were removed during transcription, and each transcript was given a number.

Inductive qualitative thematic analysis following the six-phase process described in Braun and Clarke [10] was carried out using QSR NVivo 10 [6].

Two researchers (BJH, MRG) independently coded one FGD and created an initial coding guide based on the data. The two sets of codes were then compared, and refinements were made based on discussion. Codes were then merged into themes in discussion between researchers, and a coding frame was developed. One researcher coded all transcripts (MRG). These codes were then checked independently by a second researcher (BJH), and discussion regarding further refinement to the themes and codes was conducted. In a final stage of analysis, to ensure reliability of the coding and themes, a third researcher who was independent of the current study, coded a random FGD, using the developed coding frame. This step was included to ensure that the themes and codes emerging were reliable. No substantive changes were made to the emerging themes following this step. The final themes that emerged from the analysis were then provided to members of the community (local domestic workers and key informants) during meetings arranged to elicit their feedback on themes and interpretations (i.e., member checking). Interviewer objectivity was checked through ongoing communications between researchers. This process enabled discussion towards minimizing biased interpretations of the data. Since the interviewer was a female Filipino migrant herself at the time of the study, the impact of the interviews was processed through debriefing sessions to maintain objectivity.

### Participant characteristics

In total, 22 female Filipino domestic workers participated in the FGDs. The mean age of Filipino domestic workers was 42.9 years old (*SD* = 7.47). Twelve were married, seven were separated, two were single, and one did not give an answer. Twenty were mothers, with about two children each (*M* = 2.50, *SD* = 1.47). On average, the women had been working in Macao for 4.41 years (*SD* = 5.12). Ten had only worked as an OFW in Macao, whereas the remaining 14 worked in at least one other country, notably Hong Kong and Taiwan. Of the 22, 17 were in stay-out (i.e., living in boarding houses with other domestic workers), and 5 were in stay-in (i.e., living in their employers’ home).

Among the seven key informants, six were Filipino, and one was a local Macao Chinese. Four were female, and three were male. Three were members of the Catholic Church, two were members of the Philippine Consulate General Office of Macao, and two were members of a local NGO. They participated in two separate FGDs, except for the NGO staff who were interviewed individually. The mean age of key informants was 43.43 years old (*SD* = 10.20). They had been working with Filipino domestic workers in Macao for an average of 2.79 years (*SD* = 1.68).

Theoretical data saturation occurred following the 3rd domestic worker FGD. No new themes emerged during the fourth and fifth FGDs. For the key informant interviews, members of the consulate added a new theme related to transition from tourist to migrant worker, which is the most common pathway that Filipinos become employed in Macao. Below we present the study results first by highlighting key health issues reported, followed by the social determinants of these health problems, organized by social and community networks, living and working conditions, healthcare series, housing, and the larger policy and cultural context. These themes emerged during data analysis.

## Results

### Key health problems identified

Poor physical and mental health were reported. Physical health concerns included non-communicable diseases (hypertension, diabetes) and other common illnesses including arthritis, skin rashes, colds, and fevers. Non-specific medical problems were mentioned that affected organs (lungs, heart, kidneys, gall-bladder, appendix, and stomach). Additional concerns related specifically to stress and included chronic body pain, dizziness, loss of consciousness, and extreme fatigue. Poor access to healthy and sufficient food was reported. Secondary insomnia was also commonly reported. These are illustrated in the following excerpts:

One domestic worker stated: *“Your sleep is also lacking. I’m one of them. I get dizzy as well. That’s why they say, if you’re an OFW, everything’s inadequate. Inadequate food, inadequate sleep.*”

One of the key informants from an NGO observed this as well: *“Yes, because they don’t have any, you know, vitamins in their body already. They are very weak, and they have been working and working for so many years.”*

Four themes associated with mental health problems were identified. First, stress and burnout were associated with the following reported symptoms/idioms: mind flies, inability to concentrate on job, lazy to work, difficulty sleeping, self-pity, feeling down, feeling angry, heart palpitations, thinking too much, and feeling nervous.

One domestic worker stated: *“My first employer, because it was my first time, in 2009, the effect [on me], I couldn’t sleep at night. Then I really feel pity for myself. You know that. So it’s not good, because the whole year like, I was really down, it’s not good.”*

Second, a depression-like syndrome was noted, that include the following signs and symptoms: gloomy face, looking haggard, always sad, social withdrawal, feeling lazy or lazy to work, inability to do tasks, becoming slow, and thinking of suicide. One domestic worker described a person with these problems as: *“Frowning. Carries the weight of the world. Gloomy face. Face always looks sour.”* A key informant who was a member of the Church shared that a person who shows these symptoms could not seem to feel better, *“even during prayers, she doesn’t become happy … it’s difficult to, for her to unload her problems … it’s so difficult for her to drop them.”*

Third, serious mental illness was described as “sanity gets broken,” “goes insane,” and “loses mind.” The related signs and symptoms reported for people with these issues were: stares off into space, can’t interact with others, always feels paranoid, loses self or right frame of mind, can’t hear people speak to you, can’t make decisions, gets irritated easily, and looks unkept, old, and ugly.

One domestic worker stated: *“You can’t talk to her sensibly.”*

Another domestic worker stated: *“Even if you talk to her, it’s like she [heard] nothing. Like nothing, like you didn’t say anything, like she didn’t hear anything, things like that.”*

A key informant from the consulate recalled: *“..she was really just blank and then she was really dazed.”*

Fourth, addictive behaviors were reported including drinking, smoking, and gambling. Gambling, in particular, was a significant challenge for domestic workers in Macao. Since many are already in debt, they attempt to gamble to earn money and pass the time. However, this behavior was linked by participants to problems at work, including negligence, absenteeism, job loss, loss of money to pay for daily needs and remittances. Borrowing money led to indebtedness, which in turn led to loss of friendships, stealing, and exchange sex.

One domestic worker recalled: *“She learned how to gamble, that’s why she used the money to buy things at the market to gamble. So she didn’t have anything to cook in her employer’s [house]. What she did was to go to our boarding house then, she took the rice grains there so that she has rice to cook in her employer’s [house]. The budget, she just gets eggs from the boarding house.”*

Another domestic worker said: *“They sell their bodies for sex to the foreigners”*

Table 2 provides a summary of key determinants of health, which are detailed below.

**Table 2:** Summary of themes from key informant and focus group discussions

Social determinant	Theme	Sub-themes
Social and community networks		
	Social relationships: Family	Physical separation, broken families and infidelity, problems with children, financial, and familial misconceptions.
	Social relationships: peers	Gossips and intrigues, fighting, money issues, stealing, being inconsiderate, and poor conflict management.
Work and living conditions		
	Work Environment	Strict and unreasonable standards, unpredictable and poor mood, misunderstanding due to culture and language, being treated without dignity, and controlling behavior.
	Healthcare services	Barriers to healthcare access emerged including lack of medical insurance, low trust, and language difficulties.
	Housing	Expensive housing and overcrowding.
Cultural and policy environment		
	Problems with employment agencies	High agency fees and agency exploitation.
	Inadequate labor protections	Not following labor laws, and unreasonable, unlawful terminations.
	Perceived discrimination for being a domestic worker	Discriminated by the local Chinese and by fellow Filipinos who are not employed as domestic workers.
	Geopolitical concerns	Political issues between The Philppines and China.

### Determinants of health: Social and community networks

#### Social Relationships

Difficulties across relationships with family members and peers were noted. Relationship problems with family were divided into 5 sub-themes: physical separation, broken families and infidelity, problems with children, financial, and familial misconceptions. Physical separation is a natural consequence of working as a OFW in a foreign country. Domestic workers noted an inability to take care of families back home. This is associated with an internal conflict since for many, their primary responsibility as a domestic worker is to care for another family while neglecting their own.

One domestic worker shared: *“Of course, you’re used to, with the things you do every day they’re [family] with you. And then taking care of your child and then when you arrive here, you take care of someone else.”*

Separation is also associated with sadness and loneliness. When family members back home are ill, this causes additional strain. A domestic worker said:

*“Sometimes when you feel, you feel many things, life’s difficulties, that’s when you, like you need your family more. That, and especially when you hear about a family member who gets sick, that’s when homesickness sets in.”*

Being separated also comes with other costs, as separation from marital partners is associated with infidelity. As one domestic worker put it*: “If a guy goes abroad, he becomes single again. If a girl goes abroad, she becomes single again.”*

Problems with the husband also involve child rearing. As the husband is alone to mind the children, perhaps with help from relatives, disagreements over responsibility and how a child is raised is common. For example, one domestic worker stated:

*“And the children sometimes because the mother, there, usually when she arrives here, the children left in the Philippines are neglected by the father.”*

Disagreements and martial struggles also influence the relationships that the domestic worker has with her children back home. One domestic worker shared: *“Because I left them with their father, their father had so many things to say [about me]. That’s how I.. that’s how I lost. They got brainwashed.”*

Relationships with children can be strained,

A domestic worker said:*“Yes and then they will make you feel guilty, like, you were not around when they needed you, none, they are used to not having a mother. It’s so painful.”*

These relationship challenges with children are also related to showing discipline and maintaining their position as a parent. For example, a domestic worker shared:

*“Lose their way. That’s when you like lose, like that’s when you’re a failure, that’s your failure.”*

Financial disagreements also strain family relationships. Remittances sent home are the embodiment of sacrifice for domestic workers. Once the money is sent home, domestic workers have little control over how that money is spent, and disagreements about expenditures are not easily resolved. Feelings of resentment and lack of appreciation for their sacrifice was shared by one domestic worker:

*“Like you just pick up money [from the ground], they think money comes easily (laughs) They don’t know life is difficult. My God! They really don’t know.”*

Others noted that family members might ask often for money and the amount of remittances never seems adequate. This strains the roles as noted by one domestic worker and a key informant from the consulate, respectively:

*“Sometimes I feel like an ATM machine, like that.”*

*“….the others don’t work, they just wait, right? There are those.”*

In order to gain additional remittance, some family members may use love or withdraw love and affection to manipulate domestic workers.

*"But then, he [the husband] would appease me because he has needs, right? He will appease me. “Ah he keeps on,” I said, “Ah he keeps on sending text [messages]. And like he has changed already.” Turns out, it’s followed by, “Send me some money.”*

Misconceptions may fuel these issues. Macao is a relatively richer environment, and the perception of people is that in Macao, people live a luxurious lifestyle. This is partially cultivated by domestic workers who attempt to appear as though they are living a good life aboard, motivated by protecting family members from the reality of their suffering, and to maintain their own dignity. As a result, some family members may believe they are living a *“rich life,”* the *“good life,” “just relaxing and having fun,”* and that they have a *“new family.”*

Domestic workers experience financial problems not just with regard to their nuclear families, but with their extended families as well. Akin to Filipino traditions, they are expected to share their earnings with their birth families, at times extending help even to their grandparents, nephews, and nieces. A key informant from the consulate acknowledged this cultural expectation while recounting a domestic worker’s story: *“Because she sends a different person 500 [Macau Patacas ~$65USD], another person 500 [MOP]. Here’s for her child, here’s for her mother. So how much is left with you, right?”*

To have more money to send to their families, domestic workers work more than their usual hours, either by foregoing their day-offs so that their employers will pay them extra or by finding part-time work with another employer. Having part-time work is against Macao labor law as their visas only allow them to work for the employer who sponsors them. A key informant from the Church explained: *“And of course, the government might catch them because it’s illegal and, of course, their physical [health]. They don’t have rest because they do part-time work during their rest day. Right? So for example, their rest day is Sunday, then they do part-time work, they don’t have rest day anymore.”*

To send more money, domestic workers resort to borrowing money from peers, employers, or financial companies. This can be problematic when lending terms may violate international and local labor laws, such as surrendering their passport or work permit as collateral and being charged high interest rates, which they have difficulty paying. A key informant from the consulate recounted a news article about two Filipinos arrested for lending money with unreasonable interest rates: *“Macao defines usurious rates as 10-15%. That’s against their law.”*

Relationship problems with peers were divided into 6 sub-themes: gossips and intrigues, fighting, money issues, stealing, being inconsiderate, and poor conflict management.

Gossip or *chismis* and intrigues were related to rumors spread among the Filipino community in Macao and to employers. This served to undermine support seeking, as noted by one domestic worker:

*“Because, because there are people who are, yes, they will listen to you, but they will gossip about you. Right? There are people you sometimes really trust that you tell your problems to them. But despite that, you think they sympathize with you, helping you but, the, you know how they just make up stories about you?”*

Further, these problems can lead to sullying the reputation of a domestic worker. For example, one domestic worker raised this issue: *“Sometimes when there are fights, she will go to your employer and will ask her to remove you from your job.”* A key informant from the consulate also shared: *“So like the aggrieved party said her name got destroyed with her employer, so when she came back, she didn’t have a job anymore. She just went on a vacation, then when she came back, she didn’t have a job anymore.”*

Fighting over relationships also happens. There are those who fight over romantic partners, as noted by one domestic worker:

*“Sometimes, there’s only one guy. So they will fight [over him], pull each other’s hair.”*

Fighting also occurs at the organization or peer group level. When a member is part of several groups but fails to devote equal time and effort, jealousy and fights may ensue. This was observed by a key informant who was a member of the Church:

*“If you’re a member of an organization, don’t join other organizations because if you neglect one [of the organizations], they will sulk. And you’re not the only one they are enemies with, but the entire group is their enemy because they took you away from them (laughs).”*

Issues with money were centered on borrowing and lack of repayment and non-payment of rent:*“Then when you ask for your money back, you’re the bad guy. They curse you outside.”*

Stealing is also a concern. In one extreme example, one domestic worker, living in a boarding house recounted: *“…my friend because she stole, she sold, she borrowed my passport then sold it for 5,000MOP [~$*400 USD*].”*

Being inconsiderate, especially within the boarding houses, included not sharing food, using possessions without permission, not helping with chores or making chores more difficult. This issue is exemplified when one domestic worker stated:

*“Sometimes, you did your laundry already, then someone would cook ‘tuyo’* [a type of smelly fish]*… then [the one who did her laundry] gets really angry.”*

Poor conflict management was also a salient theme. For example, a domestic worker talked about her housemate: *“She hasn’t even understood the situation, there’s a reaction, reaction, negative reaction right away. She hasn’t even understood the topic, the problem. Her reaction, it’s different right away, her reaction’s aggressive right away.”*

### Determinants of health: Living and working conditions

#### Work Environment

Problems with employers were divided into five sub-themes: strict and unreasonable standards, unpredictable and poor mood, misunderstandings due to culture and language, being treated without dignity, and controlling behavior.

Strict and unreasonable standards were perceived by participants as associated with employers’ desire to maximize the domestic workers’ time working, and this translates into long working hours. Lack of appreciation and being forced to redo work to meet these demands was also reported.

*“Like, like your every minute is paid. Like you don’t have freedom anymore, right, right?”*

There are also employers who require domestic workers to work in several houses for extended family members. For example, a domestic worker is contracted to work for their employer in their home, but the employer asks them to clean their parent’s home or wash the car of other family members. A domestic worker shared:

*“Because like me during the interview, she told me my work is only very easy, just attend to the child, just clean. So, I didn’t expect that, it’s really, they didn’t say that I have [to work on] two houses. So I’m shocked… when I signed already.”*

This is associated with always feeling pressured to work and experiencing excessive scolding and fear of employer’s reactions.

*“….because they have so many comments, that I smell, you complain a lot, you are never contented, your house is already clean, you want 100%.”*

*“Just a little from your employer, shout, you tremble already.”*

Other concerns include disputes about salary increases and providing salary on time. For example, domestic workers recalled:

*“But when she promised she will increase [the salary], she will increase my [pay], up to now it’s not”*

*“When you give my salary delayed, you don’t hear anything from me. 9-10 days after the supposed day of compensation, that’s when you give my salary.”*

Unpredictable and poor mood sets the tone for uncomfortable and precarious working conditions. This leads some domestic worker to report:

*“You know, when they make you absorber of their stress.”*

*Cultural and language issues.* Most Filipino domestic workers know little about Macao’s official languages, Chinese and Portuguese. This presents problems communicating with their employers. A key informant from the consulate mentioned: *“When you make a mistake, I think, with the accent … the meaning is different, like that. So they don’t understand each other. Maybe, the understanding of instructions is wrong, of course the employer will get mad.”*

Lack of trust is also a concern. Accusations of theft and child maltreatment are frequent. Excessive worries that domestic workers will steal from them lead to the installation of video cameras or asking a relative to watch over the domestic worker, which leads to lack of privacy. Lack of trust intensifies when employers hear news reports or gossips about domestic workers stealing or hurting children. A domestic worker recounted:

*“When they heard about a Filipina who stole something, you know, whenever they leave, the kungkong [grandfather] is there … so whenever they leave [before], they did not lock their master bedroom. But since they heard about that, they locked it already. Plus there’s a kungkong to check.”*

Being treated without dignity is manifested through the need for absolute obedience. For example, one domestic worker stated:

*“They don’t accept their mistakes, there are employers like that. When, when, uh like my first employers. “Don’t reason out.” But I reasoned out. She said, “Don’t argue with me!,” she’d say that.”*

And employers may treat the domestic worker as only employees, and not as people with their own needs, as described by one domestic worker: *“Because when you’re a helper, you’re a helper to them.”*

These circumstances with employers can lead domestic worker to suppress their emotions:

*“You know the feeling that you’re losing your humanity. But you will just swallow your saliva.”*

Controlling behavior is manifested through limited access to technology, restricted movement in the home, through food offered, allowing them to show emotions, and physical abuse.

Some domestic workers are not allowed to watch TV, use the internet, and are required to stay in the kitchen or helper’s room. Some who live with their employers are prohibited to go outside their employer’s house, as recalled by a key informant who was an NGO worker:

*“Seven days, 16 hours, inside the house of the employer. Cannot use the phone. Cannot go out. If they go out, they go with their employer, taking the car. Cannot go out by herself.”*

Dietary freedoms are also limited, especially for domestic workers who live with their employers. For example, one domestic worker stated:

*“If the food is chicken, you get the neck, you get the feet. If the food is fish, you get the tail.”*

Limitations on permissible emotions further dehumanize domestic workers. For example, one domestic worker stated:

*“Quiet. You can’t, when you’re frowning, “What’s wrong? Have a problem?” While you’re tired, you’re pissed. They want you to smile, but you’re pissed.”*

Physical abuse was also reported, as mentioned by two domestic workers:

“*Yes, then you’ll get scorched with an iron. Right, what they are holding, yes.”*

*“There are employers who will make you drink soap. My friend, before, oh my, she was really made to drink soap.”*

#### Healthcare services

Significant and notable barriers to healthcare access emerged including lack of medical insurance, low trust, and language difficulties.

Although accident insurance is compulsory for employers according to Macao labor law, comprehensive insurance that includes preventative health appointments, gynecological visits, and routine medical care is not included. The imperative to continue working in Macao to send remittances home, coupled with low wages and high medical fees, was associated with forgoing treatment and postponing medical care until they returned home, which could worsen their illnesses.

One key informant who was an NGO worker said: *“If they have rather big [physical health] issues in Macao, they may need to pay much, and they might not be able to afford.”*

A key informant from the Church commented: *“Expensive. They are actually very scared to get sick. Actually, they are scared to get sick to the extent that even if they’re already getting sick, they don’t want to see themselves as getting sick. Until they’re at the point when they couldn’t… rehabilitate or recover, what do you call that, recuperate. And then their bodies just collapse.”*

Although the law mandates insurance, it may not be clear what level of insurance is required – accidental or comprehensive – and what expenses the employer should reimburse for medical care. As one domestic worker noted:

*“It’s like that with me as well, she reimburses. For the others, they don’t. It’s up to you to undergo treatment. But, which is right, you should, you should have insurance, right?”*

Another barrier identified was pervasive lack of trust for local medical treatment. For example, one domestic worker stated “*From the local hospital. It’s rare to come out alive. The next [stop] is really the morgue, straight [to the morgue].”* A key informant from the Church recounted a domestic worker who went to the local hospital, *“Isn’t it, there was one who went to us, [she] asked for help because she had stomachache? To know about the stomachache, she needed to have an operation [in the hospital]. Of course, you’ll be scared.”* This lack of trust was associated with unwillingness to undergo treatment and a feeling that no one is available to help when illness occurs.

Communication with locals is also difficult, which then leads to problems like failure to obtain services. For instance:

A key informant who was a Church official noted that when a sick domestic worker visits a local clinic: *“For example, their employer has a doctor, they will bring her there as well. But then, what’s difficult is if they don’t understand what the ailment is. That’s due to the barrier of, the language itself. Sometimes, especially with those ailments, it’s so difficult.”*

A key informant who was an NGO worker shared: *“But there is not much practical support, especially medical care we talked about earlier, and there is a more lack of legal support. Also, difficulties in communication at governmental departments. This is because English is not our official language. You speak either Portuguese or Chinese. If you come [and] speak English, I can ignore you. This is how they think.”*

#### Housing

Current salary conditions are too low to maintain a reasonable quality of life. The average salary of a Macau person is 15,000 MOP [16]. The average salary of a domestic worker is 3000 MOP (375 USD) per month, and if they live outside the home, the additional “live out subsidy” from employers is 500 MOP (~$65USD). The average apartment rental is 10,000 MOP, which means that many domestic workers must live in crowded boarding houses, with 6–12 housemates, that is intended for a family of 3–4. For example, key informants from an NGO and the Church said, respectively:

*“Yes, it’s hard to imagine. Three or four hundred square feet…it’s unbelievable for over 10 people to live within.”*

*“Because sometimes their houses are congested so they have to go out. They have to go out. Because when they arrive at their houses, their houses are congested. You really cannot take a rest.”*

### Social determinants of health: General policy and cultural environment

#### Problems with employment agencies

High agency fees and agency exploitation of workers is also common. The fees for agencies are high, so many domestic workers are already experiencing indebtedness. Without using agencies, some domestic workers reported that it is hard to gain employment.

For example, one domestic worker stated: *“If you have money, then you’re the one who’ll get an employer (laughs).”*

A key informant from an NGO shared: *“Maybe you have heard of it somehow. Like more than 10,000 [MOP], they might [be] charge[d]…So they come to work a few months for nothing, after that, they start to really earn money.”*

#### Inadequate labor protections

Some employers circumvent Macao’s labor laws. This is facilitated by a lack of a standard labor contract, which leads to individualized contract agreements that may not be followed by employers. Although labor laws require that domestic workers are given 4 days off per month and only work in the employer’s home, employers may either be unaware of the laws or do not follow them. One common issue stated by domestic workers was that days off or leaves are not granted. For instance, a key informant from an NGO said:

*The problem is it’s not in the form of rewards. If the deal is, “If you do this, you will get that,” but it’s not like that. It’s more like, “You must not have day off if they want to work at his place.”*

For some domestic workers, the salary indicated in labor contracts are not honored. For example, one domestic worker shared: *“My friend only gets 1,900 [MOP]. 1,900 only but the contract states 4,010 [MOP]. It’s not given to her, it goes straight to her family in The Philippines, that’s why she doesn’t hold anything.”*

Finally, unreasonable, unlawful terminations, and the threat of those terminations pervade domestic worker’s experience. Summary terminations occur due to sickness. If a domestic worker has an illness, she may be fired and sent back home without warning. One domestic worker shared:

*“Because you’re scared that when your boss learns about it, you’ll get terminated. Of course, they are scared that someone’s sick.”*

Given that the domestic workers’ visas follow their employers, if there is a disruption to work, they will lose their right to remain in Macao. Domestic workers also do not receive a termination subsidy, so if their work is terminated, their salary is immediately stopped. Sometimes, terminations or resignations lead to reentry difficulties. One domestic worker suggested that this situation is dire:

*“….for us if we resign, we get banned in Macao. We will get banned.”*

Jealousy from female employers about sexual appeal and closeness with children, or being a better English speaker, also creates tension and may lead to termination.

*“And if you’re prettier than them, there, they will also ask you to leave (laughs).”*

*“That’s why the child, s/he will say, “Auntie, do, can you do, can you do my homework?” Like that. But then the mother’s there, s/he won’t ask the mother to do [the homework] because the child said the English of the mother is all wrong, like that.”*

*Perceived discrimination.* Domestic workers also perceive discrimination in Macao because they are domestic workers. They think that others look down upon them because of their job. Domestic workers perceive being discriminated by the local Chinese and by Filipinos who are residents in Macao or are employed as hotel or restaurant workers or as professionals.

For example, a domestic worker recalled: *“If they know you are tailing after your employer, you have with you the children, the employer’s children, the Filipinos, will they smile at you? No. … They will not pay attention to you if you are a domestic worker.”*

A key informant from an NGO also shared: *“Even one’s Filipino, they won’t communicate with them, talk to them. “Oh, you are a domestic helper. I am an engineer,” like that. The sense of hierarchy is quite strong.”*

*Geopolitical concerns.* Political issues also affect the treatment of domestic workers.

*“Especially when there were those who were [taken] hostage. When there were people from Hong Kong who died in Manila. There were those [Filipina domestic workers] who got terminated.”*

## Discussion

This study presents the first qualitative evaluation of the psychosocial challenges and health-related problems facing Filipino domestic workers in Macao, China. The current study utilized a qualitative approach to engage multiple stakeholders in discussions about critical health needs and determinants of health. The current study identified key health problems, and themes related to the social determinants of health, across social and community networks, living and working conditions, and the cultural and policy context in Macao, China.

The finding that domestic workers experience physical and mental health problems highlight the level of strain and vulnerability they face, compounded by the lack of access to resources, including access to basic daily necessities and healthcare. Common mental disorders including anxiety, depression, and exposures to potentially traumatic life events were noted. This is consistent with previous systematic review and other studies that note these challenges are experienced by transnational and labor migrants [12, 27, 45]. These findings further show how physical and mental health problems are pervasive, affecting their daily living, social interactions, and performance at work. These could also potentially escalate into more serious problems including suicide.

This study also found that in Macao, some domestic workers developed problematic gambling, which in turn exacerbates psychosocial and employment problems. This suggests the important role that context plays in understanding the stressors experienced and in the coping strategies that are utilized by transnational migrants. That is, within the Macao context where gambling is a major industry, gambling is an accessible and attractive. A related critical issue is the risk of HIV and other sexually transmitted infection due to exchange sex. Further research is needed to identify the burden of these diseases, especially within the context of poor healthcare availability and lack of routine and preventative medical care.

### Social determinants of health: Social and community networks

Filipino domestic workers reported problems in their social networks. Physical separation from family was linked to sadness, marital disruption, and parenting difficulties, as was found in past studies [4, 17, 30, 37]. The finding that being a breadwinner may be problematic is worth emphasizing. While providing for the family is domestic workers’ way of showing concern [34] and is a justification for being separate from one’s family [4], this study shows that family’s excessive reliance on their remittances, both in terms of families asking for too much money and being perceived only to care about money, undermines the sacrifice domestic workers are making. Although in many instances domestic workers are breadwinners, they continue to desire their traditional gender role of being caregivers even from afar [17]. This is in contrast with male migrants who are more comfortable just being breadwinners [31]. Thus, when they perceive that their families only care about remittances, domestic workers may perceive losing the important role of being a mother or caregiver. This finding can also be explained as a tendency of being a *tagasalo*, which is literally one who catches others or wanting to bear the load for one’s family out of filial piety, but at the risk of becoming burned out [3, 35].

This study found that peer relationships in the receiving country can be sources of difficulty as well, leading to distrust and social conflict. This resonates with previous studies [22, 29] showing that when experiencing adversities, social network support and social capital among Filipinos increased poor mental health. This finding reinforces the notion that being with peers who are similarly stressed and lacking control may not necessarily be good for mental health [29]. However, given the lack of alternative sources of support for foreign labor migrants, especially for low income earners like domestic workers, peers may be one of the only options for social support, and choosing not to socialize either may not be an option, or lead to social isolation.

### Social determinants of health: Living and working conditions

Problems with employers were also found, similar to previous studies [5, 37], suggesting that domestic workers are susceptible to abuse. Being asked to work long hours, without rest days, appeared to be commonplace among domestic workers. One critical distinction highlighted in this study is that domestic workers are bound by the individual policies of the household, and have few rights and protections offered in their host country. In this context, the relationship with employers takes on special significance. Employers have the authority to decide on the rules and regulations and the provision of benefits that directly influences domestic workers’ welfare. This has policy relevance which is discussed below.

Housing was a substantial issue noted in the study. Unlike domestic workers in neighboring Hong Kong, domestic workers in Macao enjoy the freedom of living outside their employer’s home. However, it is unclear to what degree this “stay-out” status confers additional detrimental health exposures due to overcrowding in boarding houses, the quality of this housing stock, and uncertain sanitation, safety, and privacy. Housing offered in employer’s homes might also not be safe, private, and suitable. Anecdotal reports suggest that domestic workers seldom have their own rooms, either co-sleep with children and elders in their care, or sleep in living rooms, or areas not appropriate for sleeping (e.g., the kitchen), and therefore lack safety and privacy.

### Social determinants of health: General policy and cultural environment

Discrimination emerged as a key potential determinant of health. Previous studies show that social and ethnic discrimination by a host society can influence the health of migrant workers [12, 22]. What is particularly noteworthy in this study was the emphasis domestic workers placed on discrimination by other Filipinos. It is revealing that a perceived class consciousness and social hierarchies lead to their ill treatment. Seen through the lens of internalized stigma, domestic workers have multiple intersecting identities, which may increase their risk for ill health.

Several key issues emerged that directly tie to enhancing policies related to labor protections in Macao. Domestic workers do not have standard work contracts, or minimum wage protections, which means that some workers engage in long work hours or “always on call” status, and may not be compensated at appropriate rates for their labor or prior agreements. Employment agencies appear unregulated, and as noted by multiple informants, agency fees increase indebtedness and limit social mobility. Working hours and employment in multiple households or with multiple employers is forbidden by the Macao labor laws.

What was revealed in the current analysis is that these laws are not followed, and monitoring and enforcement by the Macao government should be strengthened, to decrease worker exploitation, precarity, and illness. Moreover, enhancing employers’ management knowledge and skill may benefit the workers and employers alike, by reducing disagreements and setting reasonable expectations for both parties. Education about the labor laws through online or other short courses could improve adherence with existing protections. This includes the provision of standard accidental medical insurance. However, given that optimal health is achieved through preventative and routine medical services, widening the scope of coverage to include these services, along with increased access to culturally informed care should be considered.

Most domestic workers do not go through the government sanctioned process of deployment from The Philippines as an OFW, which includes attending pre-employment orientation seminars to better prepare them for working abroad (e.g., educating them about their rights, about the host country’s laws, language, and culture). While this approach to labor migration is currently legal, it increases vulnerability due to possible exploitation from employment agencies and employers. This may also lead to lack of enrolment in the Philhealth program, a social insurance scheme aimed to provide health insurance to all Filipinos, including OFWs. Accurate and timely information can enable domestic workers to set expectations about the demands of their work and better prepare them for their time abroad. Labor receiving territories like Macao might work with The Philippines Government and grassroots labor organizations to reduce employment tourism, and coordinate improved migrant services, which can improve the health and well-being of migrant workers. In addition to insurance benefits, additional medical and mental health facilities are needed that cater to the migrant workers in Macao. It should be noted that in the Macao context, few mental health services are available for local Chinese population [20], which makes improving access to these services all the more difficult for migrant workers.

This study has several important limitations. The views expressed by participants in this study may not fully represent the lived experiences of all Filipino domestic workers in China, or generalize to domestic workers living in other countries in the world (e.g., Israel). Domestic workers in China are comprised of several other nationalities, including Indonesian and Vietnamese women, and further research is needed to understand the shared and unique experiences of these groups. Staff from only one NGO was interviewed, which may limit the breadth of information gained from these interviews. Although care was taken to maintain cultural themes and local idioms, this current study may not be sufficient to fully capture local idioms of distress, and these concerns are open to further investigation. The interviewer (MRG) was able to gain rapport with interviewees; additional follow-up visits were not scheduled which may have deepened some of the current insights. The purpose of the current study was descriptive, which means establishing causal linkages between determinants and health outcomes was limited. Information was also not gathered which could aid in building a larger migration narrative, incorporating pre- and peri-migration experiences that are known to accumulate risks to migrants’ health [45]. With regard to the social determinants framework we adapted to present the study results, limited information was obtained about individual lifestyle factors, beyond stresses and long working hours. We were unable to adequately explore the suitability of housing provided within employer’s homes. Further studies are needed to systematically document these conditions. The current study also did not include employers’ perspectives. While beyond the scope of the current study, understanding employer viewpoints on hiring, managing, and living with domestic workers is critical to reducing labor disputes, enhance harmony in the household, and to identify pathways to enhance migrant worker health and well-being. Future research is planned that will elucidate this perspective.

## Conclusions

The current study demonstrates that some Filipino domestic workers experience critical mental and physical health challenges and have few pathways for adequate health care and social support. Transnational migrant domestic workers leave their families behind to seek economic opportunities. Financial resources that are sought through their migration are not sufficient to lead to upward social mobility, further compounding social stressors. In China, domestic workers experience chronic exposure to work-related stress, social stressors, and sub-optimal housing conditions – critical social determinants of health.

Epidemiological research is needed to establish evidence of the population prevalence of health issues, common mental disorders, and the various exposures that may exacerbate these issues. A respondent driven sampling study may be useful to access this hard to reach population. Policy-level analysis is also needed to evaluate the current labor laws governing domestic workers in Macao, how well these laws are implemented by employers, and how additional oversight can be implemented to improve migrant worker health, and support Sustainable Development Goal 8, Decent Work, agreed by all United Nations Member States. Multisectoral collaboration between the Macao Government, local NGOs, grassroots labor organizations, the Philippines Consulate, and local academic partners, could provide a pathway to structural policy changes, and intervention development, that may ultimately benefit this community.

## Abbreviations

FGDs: Focus Group Discussions
NGO: Nongovernmental organization
OFW: overseas Filipino workers
SAR: Special Administrative Region
